# Therapeutic Effects of Kefir Peptides on Hemophilia-Induced Osteoporosis in Mice With Deficient Coagulation Factor VIII

**DOI:** 10.3389/fcell.2022.794198

**Published:** 2022-02-18

**Authors:** Chih-Ching Yen, Yao-Wen Liu, Gary Ro-Lin Chang, Ying-Wei Lan, Yung-Tsung Kao, Shin-Nan Cheng, Wei Chen, Chuan-Mu Chen

**Affiliations:** ^1^ Department of Life Sciences, and Ph.D. Program in Translational Medicine, National Chung Hsing University, Taichung, Taiwan; ^2^ Department of Internal Medicine, China Medical University Hospita, College of Health Care, China Medical University, Taichung, Taiwan; ^3^ Ph.D. Program in Tissue Engineering and Regenerative Medicine, National Health Research Institutes and National Chung Hsing University, Taichung, Taiwan; ^4^ Department of Pediatrics, Department of Medical Research, Tungs’ Taichung Metroharbor Hospital, Taichung, Taiwan; ^5^ Division of Pulmonary and Critical Care Medicine, Chia-Yi Christian Hospital, Chiayi, Taiwan; ^6^ The iEGG and Animal Biotechnology Center, National Chung Hsing University, Taichung, Taiwan; ^7^ Rong Hsing Research Center for Translational Medicine, Taichung Veterans General Hospital, Taichung, Taiwan

**Keywords:** hemophilia A, osteoporosis, coagulation factor VIII, kefir peptides, micro-CT, osteoclast

## Abstract

Osteoporosis is a clinically prevalent comorbidity in patients with hemophilia. A preventive effect of kefir peptides (KPs) on postmenopausal osteoporosis has been proved. The aim of this study was to evaluate the therapeutic effect of KPs for the treatment of osteoporosis in coagulation factor VIII (*FVIII*) gene knockout mice (F8KO), a model of hemophilia A. In this study, male F8KO mice at 20 weeks of age were orally administered different doses of KPs for 8 weeks. The therapeutic effects of KPs were shown in the femoral trabeculae and the 4^th^ lumbar vertebrae, which increased the trabecular bone mineral density (BMD), bone volume (Tb.BV/TV), and trabecular number (Tb.N) and decreased the trabecular separation (Tb.Sp), and they were also observed in the femoral cortical bones, in which the mechanical properties were enhanced in a dose-dependent manner. Characterization of receptor activator of nuclear factor κB ligand (RANKL), osteoprotegerin (OPG), and interleukin 6 (IL-6) demonstrated that the serum RANKL/OPG ratio and IL-6 levels were significantly decreased in the F8KO mice after the KP treatment. Tartrate-resistant acid phosphatase (TRAP) staining of mature osteoclasts indicated that the therapeutic effect of KPs in F8KO mice was associated with the functions of KPs to inhibit RANKL-induced osteoclastogenesis by reducing serum RANKL/OPG ratio and IL-6 secretion. The present study is the first to address the potentials of KPs for the treatment of hemophilia-induced osteoporosis in mice and it also provides useful information for the application of KPs as a complementary therapy for the treatment of osteoporosis in hemophilic patients.

## 1 Introduction

Hemophilia is a mostly X-linked genetic disease in which bleeding cannot be stopped normally. The incidence of hemophilia is approximately 1 in 10,000 births, and more than 400,000 estimated patients are affected worldwide based on the World Federation of Hemophilia survey (www.wfh.org). There are two main types of hemophilia: hemophilia A, which is caused by a deficiency of coagulation factor VIII (FVIII) and accounts for 80–85% of the total hemophilia population, and hemophilia B, which is caused by a deficiency of coagulation factor IX (Srivastava et al., 2013). With proper treatment and improved medical management, the mortality rates for hemophilic patients have declined substantially. However, some underestimated comorbidities, such as osteoporosis, arthropathy, and sarcopenia, have made the prognosis of hemophilia more difficult.

Osteoporosis is a systemic bone disorder characterized by low bone mineral density (BMD) and deteriorated bone microarchitecture, and it leads to increased risks of bone fragility and fracture. Many studies have indicated that osteopenia (low BMD) and osteoporosis are prevalent in patients with hemophilia (PWH). In a previous study of young children with hemophilia, Tlacuilo-Parra *et al.* reported that 35% of them were diagnosed with low lumbar spine BMD (Tlacuilo-Parra et al., 2011). In a recent cross-sectional study of adult PWH, Kiper *et al.* reported that 34.8% of patients younger than 50 years had low BMD and 66.6% of patients older than 50 years had osteoporosis (Kiper Unal et al., 2017). In a retrospective study of PWH ≥18 years, Ulivieri *et al.* found that 74 and 54% of the patients exhibited reduced BMD at the femur and the lumbar spine, respectively (Ulivieri et al., 2018). These studies suggested that osteoporosis in PWH is underestimated and that the bone health of PWH should be properly managed because it may aggravate the diseases associated with hemophilia and affect the physical and mental health of patients.

Prophylaxis with factor VIII replacement therapy, regular weight-bearing exercise, adequate calcium and vitamin D supplementation, and fall prevention are usually recommended to promote bone health and prevent low BMD in PWH (Kempton et al., 2015). If pharmacological treatment of osteoporosis in PWH is needed, then the treatment approach is guided by medications to treat osteoporosis in the general population. Most current medications to treat osteoporosis fall into the category of antiresorptives. Bisphosphonates (BPs) (alendronate, ibandronate, etc.) are the first-line antiresorptive agents to prevent and treat osteoporosis in postmenopausal women. However, only one clinical trial using ibandronate has been reported in PWH thus far, and the results demonstrated that ibandronate was well tolerated and that oral administration of monthly 150 mg ibandronate for 12 months led to an increase in lumbar BMD and reduced bone resorption in a cohort of PWH (mean age 43.5 years) (Anagnostis et al., 2013). Teriparatide, a recombinant human parathyroid hormone 1–34, is an expensive, and active anabolic agent to treat patients with severe osteoporosis. Treatment with teriparatide has been shown to stimulate the maturation of circulating osteoblast precursors (D'Amelio et al., 2012) and reduce vertebral fracture risks (Napoli et al., 2018; Díez-Pérez et al., 2019). Long-term use of BPs is associated with an increased risk of osteonecrosis of the jaw (ONJ) (Lee et al., 2013; Chiu et al., 2014), and BP can reside in the bone with an estimated half-life of 10–20 years; however, whether any adverse effects occur has not been clarified (Khan et al., 1997). Furthermore, the incidence of medication-related ONJ in patients with underlying malignant diseases (cancer) taking BPs can reach 15%, whereas this value is only 0.01% in patients with osteoporosis (Mücke et al., 2016). The choice and decision of which medication to use depends mostly on its side effect profile and should be undertaken by consulting with an experienced clinician.

Kefir is a fermented dairy product that can be traced back to ancient Caucasus tribes, and it is produced from complex symbiotic grains that mainly contribute lactic acid bacteria, yeasts, and their metabolic products. Kefir-derived products, such as peptides, polysaccharides, and short-chain fatty acids, are subjects of great interest in Western scientific communities due to their health-promoting properties, including antimicrobial, anticancer, antiallergenic, immunomodulation, lactose and cholesterol metabolism, gastrointestinal health, and wound healing properties (Bourrie et al., 2016; Fiorda et al., 2017; Rosa et al., 2017). Our previous investigations using mouse and rat models of postmenopausal osteoporosis revealed the bone-protective efficacy of kefir peptides (KPs) (Chen et al., 2015). We found that the loss of bone mass was prevented and the skeletal microarchitecture and mechanical properties were improved in ovariectomized mice or rats after 12 weeks of oral administration of KPs at different dosages (164, 328, and 648 mg/kg/day), and the bone-protective extent of KPs displayed a dose-dependent effect and was comparable to that of the first-line antiresorptive agent alendronate (Chen et al., 2015; Tu et al., 2020). In a controlled, parallel, double-blind clinical trial of 65 osteoporosis patients, we demonstrated that the baseline turnover and the 6-month BMD change were significantly improved among the patients receiving KPs (1,600 mg KPs +1,500 mg CaCO_3_) compared to those receiving the placebo (1,500 mg CaCO_3_) (Tu et al., 2015).

Based on our previous success in OVX models and clinical trials, the present study used an animal model of *FVIII* knockout (F8KO)-induced hemophilia to evaluate the therapeutic efficacy of KPs in the treatment of osteoporosis in PWH. Initially, we analyzed the femoral bones of the F8KO mice at the age of 20 weeks to confirm the incidence of osteoporosis and then orally administered different doses of KPs for 8 weeks. Microcomputed tomography (µ-CT) for bone microarchitecture (BMD, Tb.BV/TV, Tb.N, and Tb.Sp), nanoindentation for mechanical properties (hardness and elastic modulus), and serum markers for bone remodeling (IL-6, RANKL/OPG ratio) were applied for extensive evaluations.

## 2 Materials and Methods

### 2.1 Kefir Peptide Preparation

The KPs powder (KEFPEP^®^) used in this study was provided by Phermpep Biotech. Co., Ltd. (Taichung, Taiwan) as described previously (Chen et al., 2015). The peptide content was determined to be 23.1 g/100 g by the O-phthalaldehyde (OPA) method using triglycine as a standard of calibration (Tu et al., 2015; Chen et al., 2020; Tung et al., 2020).

### 2.2 Animals and Experimental Design

The experimental procedures and animal handling were approved by the Institutional Animal Care and Use Committee of National Chung Hsing University, Taiwan (IACUC 103–100). A total of 11 male wild-type (WT) mice (C57BL/6J) and 37 male *FVIII* knockout (F8KO) mice (129S4-F8tm1Kaz/J) with FVIII levels of <1% were used in this study. WT and F8KO mice were purchased from BioLASCO Taiwan Co., Ltd. (Taipei, Taiwan) and Jackson Laboratory (Farmington, CT, USA), respectively. During the experimental period, all mice were housed in a room with an individual ventilation cage system (IVC) and maintained at 24–25°C and 50–60% humidity with a 12-h light/dark cycle, and a standard SPF chow diet (#1324–10SPF, Altromin, Germany) and sterile drinking water were provided *ad libitum*.

At the age of 20 weeks, blood was collected from WT and F8KO mice (each *n* = 5) to measure the FVIII activity and coagulation time and then sacrificed first to obtain the femoral bones for histopathological and μ-CT analyses. The other WT mice (*n* = 6) and F8KO mice (*n* = 32) were divided into five groups according to the different treatments: 1) WT (H_2_O; *n* = 6), 2) mock (H_2_O; *n* = 8), 3) KL (low-dose of KPs, 164 mg/kg body weight per day; *n* = 8), 4) KM (medium-dose of KPs, 328 mg/kg body weight per day; *n* = 8), and 6) KH (high-dose of KPs, 656 mg/kg body weight per day; *n* = 8). KPs were dissolved in H_2_O and administered through oral gavage for 8 weeks. Mouse body weight and food intake were recorded every week. At the end of the study, mice were anesthetized by intraperitoneal injection of 2.5% avertin (2,2,2-tribromoethanol; Sigma–Aldrich, St. Louis, MO, USA), blood was collected by orbital sinus sampling, and then the lumbar vertebrae and bilateral femoral bones were removed. The lumbar vertebrae and left femoral bones were immersed in 10% formalin for further characterization, and the right femoral bones were used for the isolation of bone marrow cells.

### 2.3 Measurement of FVIII Activity and Blood Coagulation Time

Mice were anesthetized and 90 μl of blood was collected and mixed with 10 μl of 3.2% sodium citrate. Citrated blood was added to a Coag Dx Analyzer (IDEXX, Westbrook, Maine, USA) for the activated partial thromboplastin time (aPTT) test. FVIII activity was measured by an FVIII Chromogenic Assay kit (Siemens, Marburg, and Germany) according to the manufacturer’s manual instructions.

### 2.4 Histopathological Analysis

To prepare femur tissue sections for hematoxylin and eosin (H&E) staining, the femur bones were decalcified in Decalcifier I^®^ solution (Leica Microsystems Inc., Buffalo Grove, IL, USA), dehydrated in a series of 50, 60, 70, 80, 90 and 100% ethanol, embedded in paraffin and longitudinally sectioned at 2–3 μm (Chen et al., 2021; Wang et al., 2021). The femur tissue sections were submitted to H&E staining using a Sakura model DRS-60A automatic slide stainer (Tissue-Tex DRS, Sakura, and Japan). The area of trabecular bones in the H&E stain images were quantified using ImageJ software. In addition, TRAP staining was also performed using a Leukocyte Acid phosphatase kit (#387A, Sigma–Aldrich) according to the manufacturer’s instructions.

### 2.5 Microcomputed Tomography

The 4^th^ lumbar vertebrae and left femur bone were used to analyze the trabecular and cortical bone parameters with a high-resolution μ-CT scanner (SkyScan 1,174, SkyScan, Aartselaar, and Belgium). Each sample was scanned at a resolution of 8 μm, rotation step of 0.3°, voltage of 50 kV, amplitude of 800 μA, exposure of 2,500 milliseconds, and reconstruction angular range of 182.7° (Chen et al., 2015). The mineralized bone phase of each resulting image was extracted using a fixed threshold and a low-pass filter to remove noise. A total of 487 two-dimensional (2D) images in each sample (a thickness of 4 mm of lumbar vertebrae or distal femur epiphysis) were reconstructed to obtain its three-dimensional (3D) image. In each 2D image, the trabecular bone was isolated from the cortical bone by manual contouring analysis. From the volume of interest (VOI), the bone mineral density (BMD), bone volume/total volume (BV/TV), trabecular number (Tb.N), trabecular thickness (Tb.Th), and trabecular separation (Tb.Sp) were obtained (Tu et al., 2020).

### 2.6 Nanoindentation

Nanoindentation was used to evaluate how KPs change the mechanical properties of cortical bones. To prepare the samples for nanoindentation, the bones were embedded in epoxy resin (Struers Inc., Cleveland, OH, USA), and then the surfaces of the embedded bone samples were polished by a milling machine. Nanoindentation was performed using a nanoindenter (Tribolab, Hysitron Inc., Eden Prairie, MN, USA) equipped with a Berkovich diamond indenter (tip radius 50 nm). Each bone was indented from the outer side to the inner side (near the bone marrow), with a total of 10 indents. The parameter settings of the instrument were obtained from our previous studies (Chang et al., 2011; Wang et al., 2013). The mechanical elasticity and hardness of the cortical bones were calculated according to the indentation load-depth curves and the Oliver-Pharr relation.

### 2.7 Measurements of Serum Bone Markers and Cytokines

The following bone markers and proinflammatory cytokines in mouse serum were measured using commercially available kits according to the manufacturer’s manual instructions: alkaline phosphatase (ALP) (Catalog #K412–500, BioVision, Milpitas, CA, USA), osteocalcin (OC) (Catalog #SEA471Mu, USCN Life Science Inc., Wuhan, China), cross-linked C-telopeptide of type I collagen (CTX1) (Catalog #CEA665Mu, USCN Life Science Inc.), osteoprotegerin (OPG) (Catalog #MOP00, R&D Systems Inc., Minneapolis, MN, USA), receptor activator of nuclear factor κ-B ligand (RANKL) (Catalog #MTR00, R&D Systems Inc.), interleukin-1α (IL-1α) (Catalog #ab113344, Abcam, Cambridge, MA, USA), IL-1β (Catalog #ab108866, Abcam), IL-6 (Catalog #ab100712, Abcam) and tumor necrosis factor-α (TNF-α) (Catalog #ab208348, Abcam). The measurements were conducted by an automated microplate reader (Tu et al., 2021).

### 2.8 *In vitro* Osteoclast Differentiation

The right femoral bone was used for the isolation of bone marrow cells. Briefly, the removed bones were washed with 70% ethanol for a few seconds and then immersed in Dulbecco’s phosphate-buffered saline (D-PBS) (Grand Island, NY, USA). The two ends of the epiphysis were cut off, and the cells were flushed from the bone marrow cavity with α–MEM (Sigma–Aldrich). The marrow content was passed through a 70-μm mash and sequentially suspended in RBC lysis buffer (0.15 M NH_4_Cl, 10 mM NaHCO_3_, 0.1 mM EDTA, pH 7.2–7.4), D-PBS, and complete α–MEM growth media (α-MEM containing 10% fetal bovine serum, 100 U/ml penicillin, 100 mg/ml streptomycin). The cells (8 × 10^5^ cells/cm^2^) were inoculated and incubated at 37°C and 5% CO_2_ for 24 h. Nonadherent cells were removed, and adherent bone marrow cells were cultured in fresh complete α–MEM growth media supplemented with RANKL (50 ng/ml) and M-CSF (25 ng/ml) for osteoclast differentiation. The cultural media were replaced every 3 days. On the 15^th^ day, the differentiated osteoclasts were assessed by TRAP staining.

In addition, the effect of KPs was also tested in the culture of bone marrow macrophages (BMMs) (Weischenfeldt and Porse, 2008), which were prepared from the femurs of 5-week-old wild-type mice. The marrow contents of femurs were flushed and cultured overnight in complete α–MEM growth media containing M-CSF (25 ng/ml). Nonadherent cells were transferred to new tissue plates to culture stroma-free bone marrow cells. After 3 days, the adherent cells were harvested as BMMs, which were cultured on 96-well culture plates with fresh medium containing M-CSF and RANKL (50 ng/ml) and various concentrations of KPs. The culture medium was refreshed once on the 3^rd^ day. On the 5^th^ day, TRAP staining was performed. The TRAP-positive multinucleated cells (≥3 nuclei) were considered as mature osteoclasts.

### 2.9 Statistical Analysis

The results are presented as the mean ± SEM (*n* = 6–8) and were graphed using GraphPad Priam software version 6.0. Statistical analysis was performed using IBM SPSS Statistics software version 20. Group differences were examined based on a one-way ANOVA and Duncan’s post hoc test, and significant differences (*p* < 0.05) are indicated by * vs. WT group, # vs. mock group.

## 3 Results

### 3.1 F8KO Mice Develop Osteoporosis at the Age of Twenty Weeks

**FIGURE 1:**
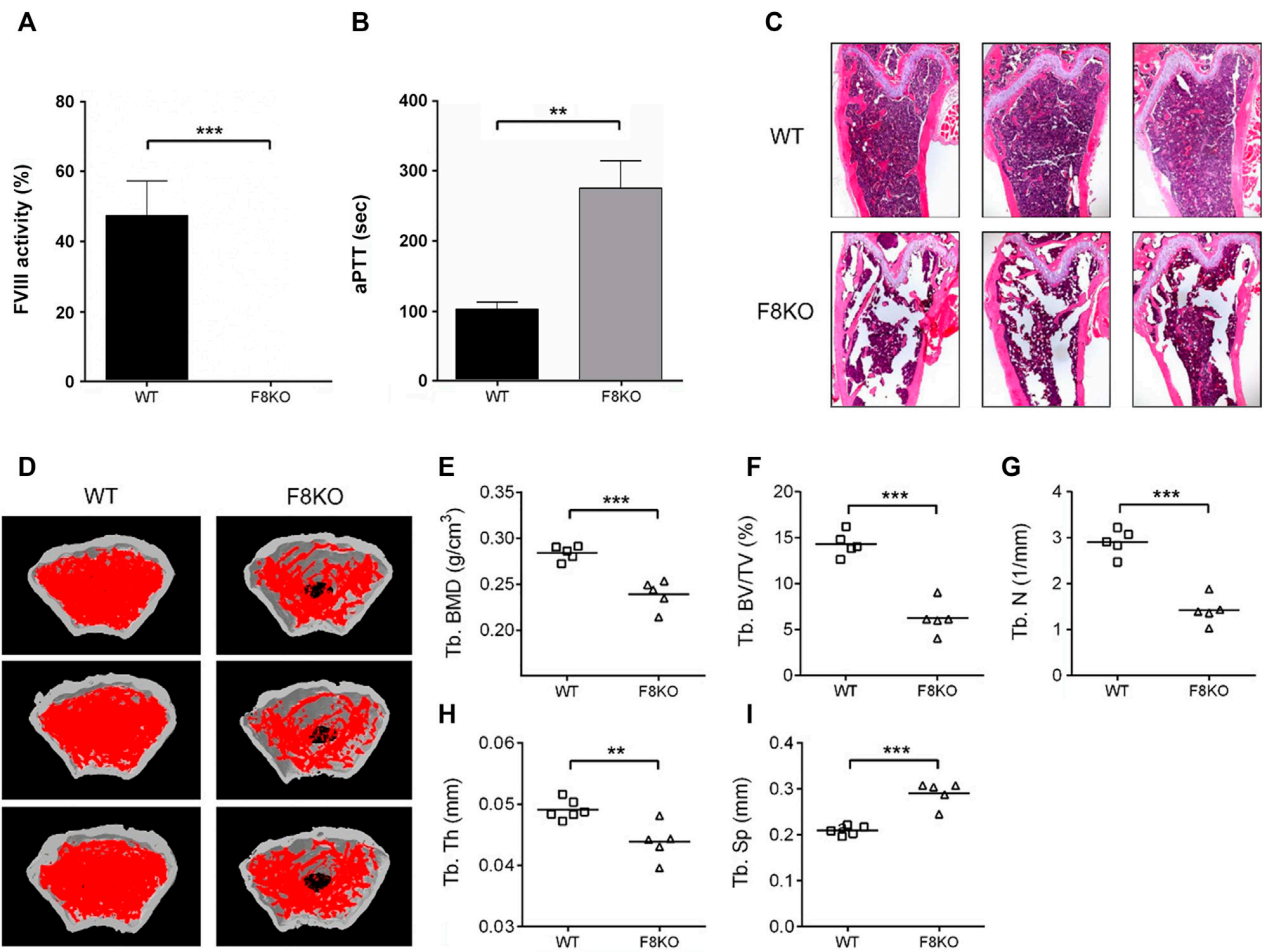
Comparison of blood coagulation activity and femoral structures between *FVIII* knockout mice (F8KO) and C57BL/6J wild-type mice (WT) at 20 weeks of age. **(A)** Blood FVIII activity measurements of male F8KO (*n* = 5) and WT (*n* = 5) mice were performed using an FVIII Chromogenic Assay kit. **(B)** Activated partial thromboplastin time (aPTT) tests of male F8KO (*n* = 5) and WT (*n* = 5) mice were performed using a Coag Dx Analyzer. **(C)** Hematoxylin and eosin (H&E) staining of femur tissue sections of male F8KO and WT mice for bone histopathological analysis. **(D)** Microcomputed tomography (μ-CT) analysis was performed to characterize femoral architectural structures. Representative images of femur trabecular morphology plotted in red. The quantitative μ-CT results of **(E)** trabecular bone mineral density (Tb.BMD), **(F)** trabecular bone volume/total volume (Tb.BV/TV), **(G)** trabecular number (Tb.N), **(H)** trabecular thickness (Tb.Th), and **(I)** trabecular separation (Tb.Sp) were shown as indicated (**p < 0.01, ***p < 0.001 vs. WT group).

Initially, blood was collected from 5 WT and 5 F8KO mice at the age of 20 weeks to measure the FVIII activity and coagulation time and then sacrificed to obtain the femoral bones for histopathological and μ-CT analyses. As shown in Figure 1, FVIII activity was significantly reduced to <1% in the F8KO mice compared with the normal WT mice (*p* < 0.001; Figure 1A), and the blood coagulation time of the F8KO mice significantly increased from 102 to 300 sec (*p* < 0.01; Figure 1B). Histopathological H&E staining (Figure 1C) and femoral μ-CT images (Figure 1D) revealed a significant loss of trabecular bone in the F8KO mice compared with the WT mice. The average trabecular BMD of the F8KO mice was 0.239 g/cm^3^, thus accounting for a 15.8% reduction compared to the WT mice (0.284 g/cm^3^) (*p* < 0.001) (Figure 1E). With regard to the changes in the bone microarchitecture, the average bone volume (Tb.BV/TV) was reduced by 56.1% in the F8KO mice (F8KO: 6.28% vs. WT: 14.31%) (*p* < 0.001) (Figure 1F), and the average bone number (Tb.N) was reduced by 60% in the F8KO mice (F8KO: 1.4 mm^−1^ vs. WT: 2.9 mm^−1^) (*p* < 0.001) (Figure 1G), and the average bone thickness (Tb.Th) was reduced by 10.2% in the F8KO mice (F8KO: 0.044 mm vs WT: 0.049 mm) (*p* < 0.01) (Figure 1H); in contrast, the average bone separation (Tb.Sp) increased 38.8% in the F8KO mice (F8KO: 0.29 mm vs WT: 0.21 mm) (*p* < 0.001) (Figure 1I). These data confirmed that the F8KO mice developed osteoporosis at the age of 20 weeks.

### 3.2 Effects of KP Treatment on Bone Histopathological and Architectural Changes

**FIGURE 2:**
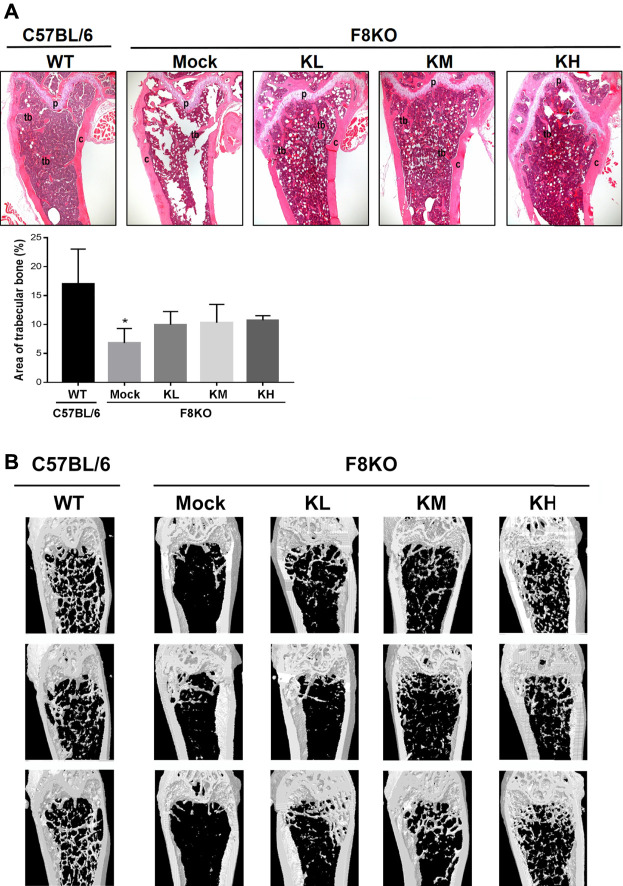
Femur bone histopathological and microarchitectural changes in osteoporotic F8KO mice after kefir peptide (KP) treatment. **(A)** Representative H&E staining images of femur vertical sections of male F8KO mice after treatment with different dosages of KPs for 8 weeks (*n* = 8). C57BL/6J wild-type (WT) mice were used as a normal control. The area of trabecular bone in the H&E images were quantified using ImageJ software as indicated. Tb, trabecular bone; C, cortical bone; P, growth plate. *p < 0.05 vs WT group. **(B)** Representative μ-CT 3D images of distal femur vertical sections of male F8KO mice after different dosages of KP treatment for 8 weeks (*n* = 8). The C57BL/6J wild-type (WT) group indicates normal trabecular bone morphology in the distal femur, and the F8KO mock group indicates server trabecular bone loss as a hemophilia-induced osteoporotic mouse. KL: low-dose KP-treated group, 164 mg/kg/day; KM: medium-dose KP-treated group, 328 mg/kg/day; KH: high-dose KP-treated group, 656 mg/kg/day.

As shown in Figure 2A, histological examination of the femur bones revealed no histopathological changes in the WT group while bone specimens from the F8KO mice without treatment showed significant trabecular bone loss. After 8 weeks of KP treatment, specimens from F8KO mice that received different doses of KP treatment showed significant recovery of trabecular bones and exhibited a comparable morphology with those of normal WT mice. The quantitative data of the areas of trabecular bones in the H&E images from each group were consistent with these findings (Figure 2A). In the μ-CT 3D image observation, we also demonstrated that the loss of trabecular bone in the femur of the F8KO mice was successfully recovered after 8 weeks of KP treatment in a dose-dependent manner (Figure 2B). These results demonstrated that oral administration of KPs inhibited osteoporosis and recovered the lost bone structure in the F8KO mice.

### 3.3 Effects of KP Treatment on Bone Mineral Density and Bone Parameters

**FIGURE 3:**
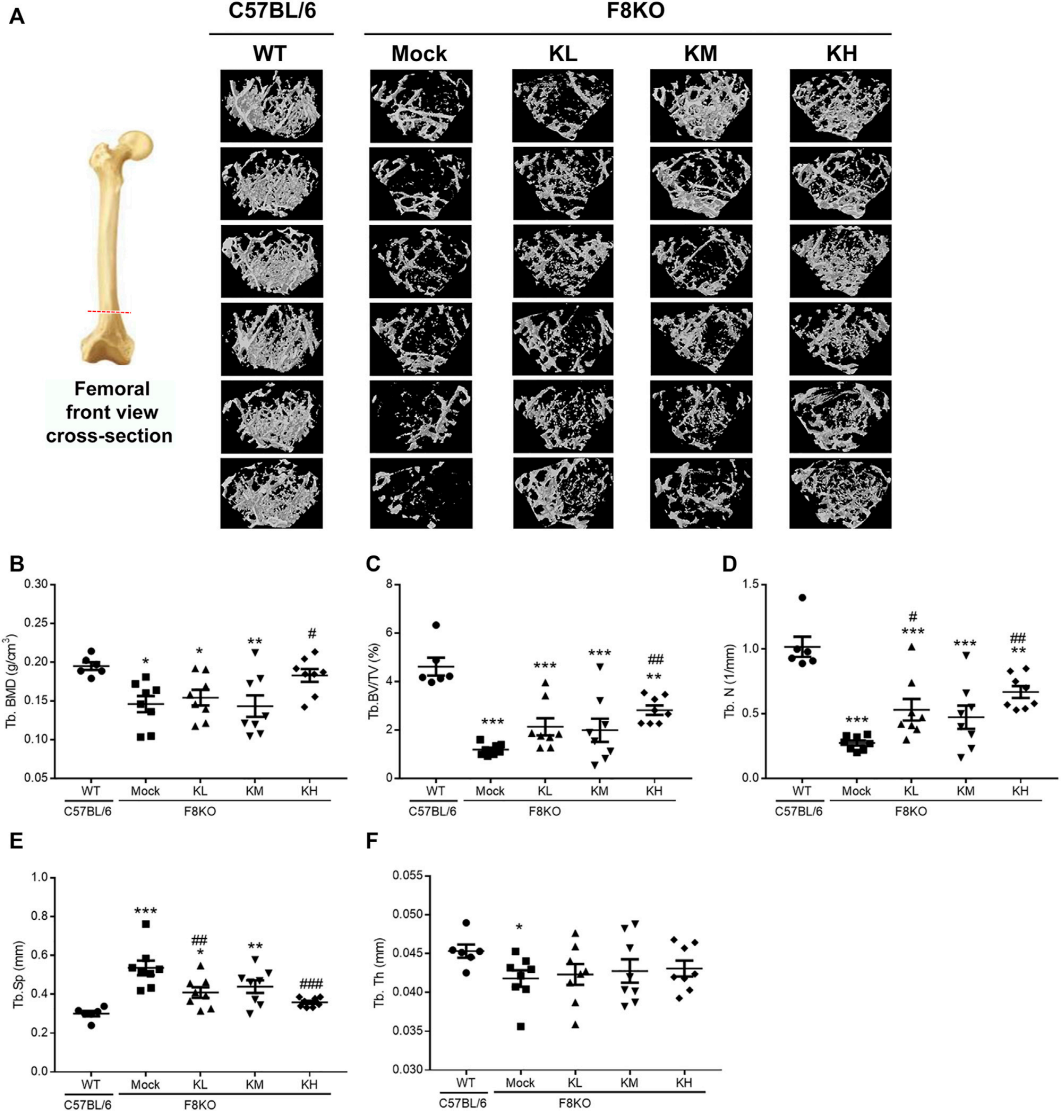
Effects of KP treatment on femoral trabecular bone. F8KO mice at 20 weeks of age were orally administered different dosages of KPs for 8 weeks (*n* = 8). At the end of treatment, the mice were sacrificed and left femur bones were analyzed by μ-CT to characterize the changes in bone microarchitectural parameters. **(A)** Representative trabecular morphological images of transverse-sectional distal femur bone. The quantitative μ-CT results for **(B)** Tb.BMD, **(C)** Tb.BV/TV, **(D)** Tb.N, **(E)** Tb.Sp, and **(F)** Tb.Th. *p < 0.05, **p < 0.01, and ***p < 0.001 vs. WT group; ^#^ *p* < 0.05, ^##^ *p* < 0.01, and ^###^ *p* < 0.001 vs. mock group.

To investigate whether KPs can improve bone mass and cause changes in the microarchitecture of the distal femur in the F8KO mice, a μ-CT analysis was performed, as shown in Figure 3A for the femoral front view and the cross-section of trabecular images. Compared to the WT mice that had dense cancellous bone, the F8KO mice that received mock treatment had relatively less cancellous bone. However, the F8KO mice that received different dosages of KPs exhibited a recovered trabecular bone network, which seemed to be positively correlated with the given dosage of KPs (Figure 3A).

The mock group had a markedly reduced trabecular BMD (0.146 g/cm^3^) compared with the normal WT group (0.195 g/cm^3^). After 8 weeks of treatment with KPs, the trabecular BMD was 0.154 g/cm^3^ in the KL group, 0.143 g/cm^3^ in the KM group, and 0.183 g/cm^3^ in the KH group. The results showed that high-dose KP treatment significantly increased the trabecular BMD of the distal femur by 125% compared to that of the mock group (*p* < 0.05; Figure 3B).

Consistent with Figure 1, after 8 weeks of treatment, the trabecular microarchitecture in the mock group showed significant changes in the levels of Tb. BV/TV, Tb.N, Tb.Sp, and Tb.Th (1.19%, 0.27 mm^−1^, 0.54 mm, and 0.042 mm, respectively) compared with the WT group (4.61%, 1.02 mm^−1^, 0.30 mm, and 0.045 mm, respectively, *p* < 0.05 or *p* < 0.001) (Figures 3C–F). Treatment with different doses of KPs in the KL, KM, and KH groups increased the levels of Tb.BV/TV (2.13, 1.99, and 2.82%, respectively, Figure 3C) and Tb.N (0.53, 0.47, and 0.67 mm^−1^, respectively, Figure 3D), decreased the levels of Tb.Sp (0.41, 0.44, and 0.36 mm, respectively, Figure 3E), and caused slight changes in Tb.Th (0.042, 0.043, and 0.043 mm, respectively, Figure 3F). Thus, treatment with KPs restored the F8KO-induced changes in the trabecular microarchitecture, and the changes in the KH group reached statistical significance.

### 3.4 Effects of KP Treatment on the Mechanical Properties of Cortical Bones

**FIGURE 4:**
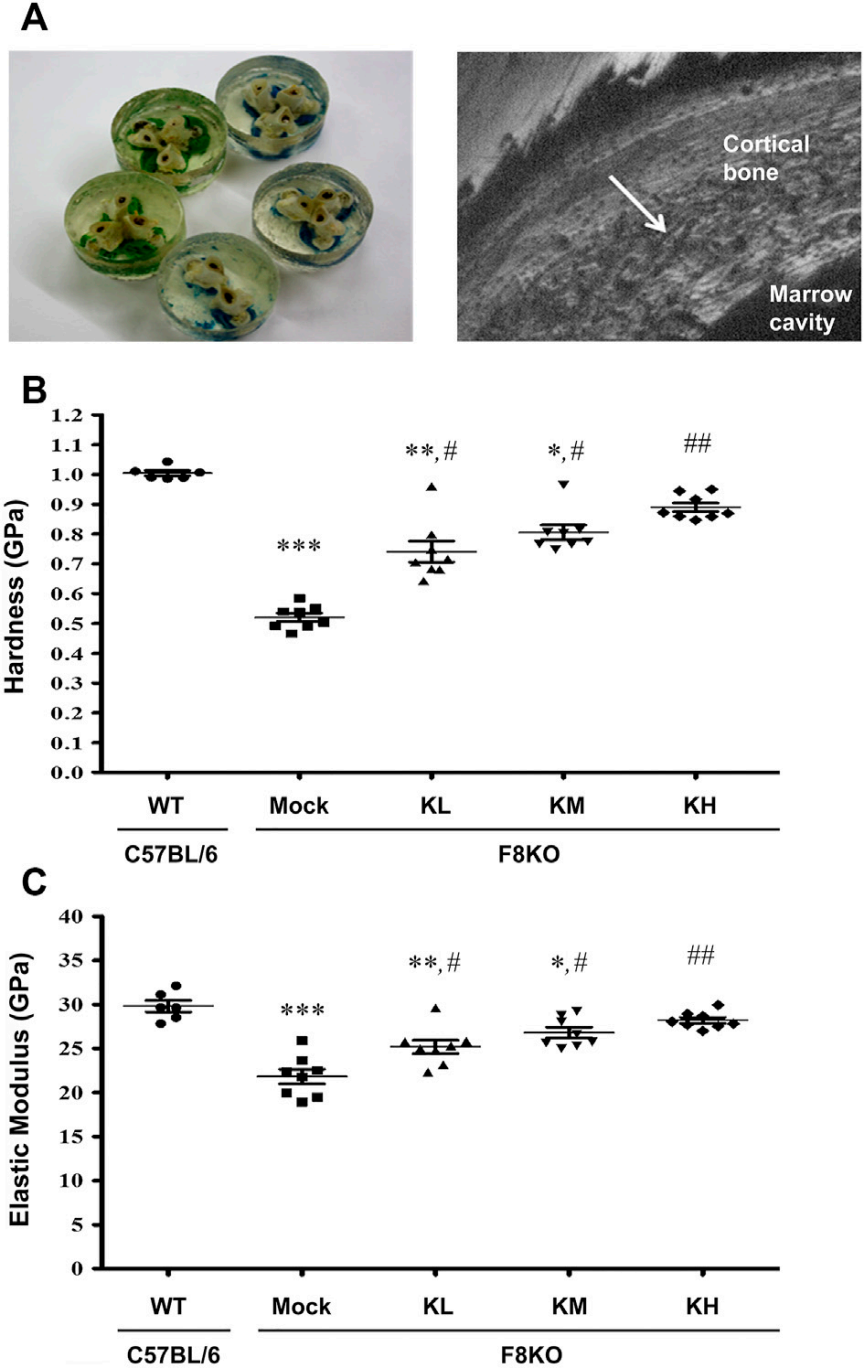
Effects of KP treatment on the mechanical properties of femoral cortical bone. **(A)** Transverse-dissected distal femur bones were embedded in resin (*left*), and a diamond indenter was used to microindent the polished surfaces of the cortical bone from the outer side to the inner side for 10 points by nanoindentation, as shown in a scanning electronic microscopy image (*right*). The quantitative results exhibited the mechanical properties of **(B)** hardness and **(C)** elastic modulus. *p < 0.05, **p < 0.01, and ***p < 0.001 vs. WT group; ^#^ *p* < 0.05, ^##^ *p* < 0.01 vs. mock group.

Figure 4 shows the change in the mechanical properties of cortical bone in different groups by nanoindentation analysis. Distal femoral bones were embedded in resin, and a diamond indenter was used to indent the polished cortical surfaces from the outer to the inner side (Figure 4A). The cortical hardness (Figure 4B) and elastic modulus (Figure 4C) in the F8KO mice receiving mock treatment were dramatically reduced to 0.52 and 21.8 GPa, respectively, compared to the WT mice (1.00 and 29.8 GPa, respectively, *p* < 0.001). After 8 weeks of treatment with different dosages of KPs, the cortical hardness and elastic moduli in the F8KO mice significantly increased to 0.74/25.2 GPa in the KL group (*p* < 0.05), 0.81/26.8 GPa in the KM group (*p* < 0.05), and 0.89/28.2 in the KH group (*p* < 0.01). Thus, oral administration of KPs significantly improved the mechanical properties of cortical bone in the F8KO mice.

### 3.5 Effects of KP Treatment on the Lumbar Vertebrae

A µ-CT analysis was performed on the 4^th^ lumbar vertebrae of the mice. As shown in Supplementary Figure S1A, the mock F8KO mice had fewer trabeculae than the WT mice. Although not obvious in appearance, the trabeculae were improved in the F8KO mice treated with KPs. Morphometric results showed that the KP treatments did not cause significant change in lumbar Tb.BV/TV ratio (Supplementary Figure S1B) but resulted in 4.6, 6.3, and 7.4% increases in lumbar Tb.N (Supplementary Figure S1C) and 6.9, 7.9, and 10.1% reductions in lumbar Tb.Sp (Supplementary Figure S1D) in the KL, KM, and KH groups, respectively, compared to the mock group. The changes with high-dose KP treatment were statistically significant (*p* < 0.05) and comparable to the WT mice.

#### 3.5.1 Effects of KP Treatment on Serum Bone Turnover Markers

**FIGURE 5:**
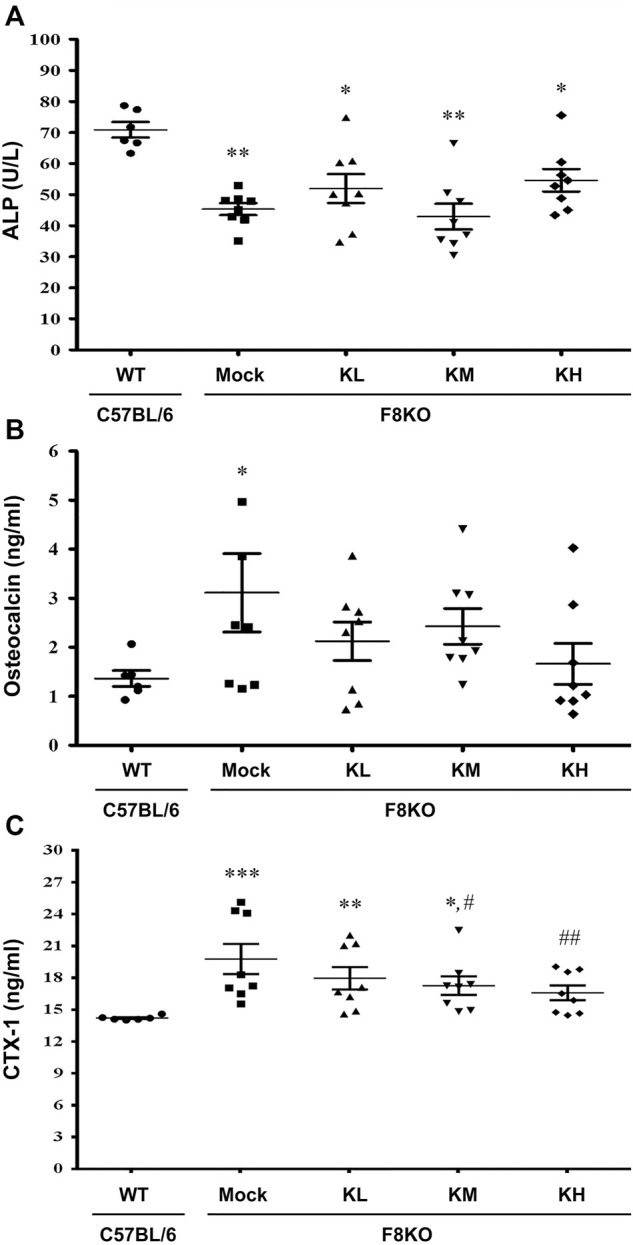
Effects of KP treatment on serum bone turnover markers. F8KO mice at 20 weeks of age were orally administered different dosages of KPs for 8 weeks (*n* = 8). At the end of treatment, mouse blood was collected for bone turnover marker detection. The quantitative data revealed the levels of **(A)** alkaline phosphatase (ALP), **(B)** osteocalcin (OC), and **(C)** C-telopeptide of type I collagen (CTX1) for the KP-treatment groups with different dosages. *p < 0.05, **p < 0.01, ***p < 0.001 vs. WT group; ^#^ *p* < 0.05, ^##^ *p* < 0.01 vs. mock group.

**FIGURE 6:**
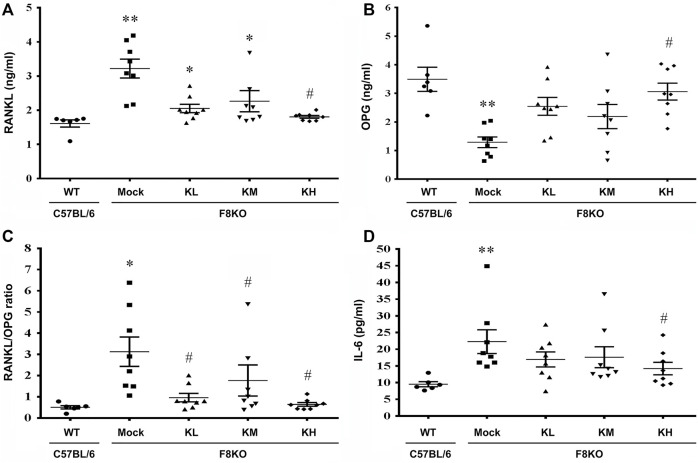
Effects of KP treatment on the serum RANKL/OPG ratio and proinflammatory IL-6 cytokine levels. F8KO mice at 20 weeks of age were orally administered different dosages of KPs for 8 weeks (*n* = 8). At the end of treatment, mouse blood was collected for bone resorption and bone formation marker detection. The quantitative data show the levels of **(A)** receptor activator of nuclear factor κ-B ligand (RANKL), **(B)** osteoprotegerin (OPG), **(C)** RANKL/OPG ratio, and **(D)** interleukin-6 (IL-6) in the KP-treated groups at different dosages. *p < 0.05, **p < 0.01 vs. WT group; ^#^ *p* < 0.05 vs. mock group.

Serum biochemical markers of bone formation (ALP and OC) and bone resorption (CTX-1) were analyzed in this study. As shown in Figure 6, a significant decrease in ALP (*p* < 0.01) and significant increases in OC (*p* < 0.05) and CTX-1 (*p* < 0.001) were measured in the mock F8KO mice compared to the WT mice. The treatments increased the ALP (Figure 5A) and reduced the OC (Figure 5B) in the groups of F8KO mice receiving different doses of KPs, although these changes did not reach statistical importance compared to the mock group. CTX-1 in the F8KO mice receiving KPs decreased, but only the treatment with a high dose of KPs led to statistically significance difference compared to the mock group (*p* < 0.01; Figure 5C).

#### 3.5.2 Effects of KP Treatment on the Serum RANKL/OPG Ratio

At 20 weeks of age, oral administration of KP or mock (H_2_O) treatment was provided to the WT and F8KO mice. As shown in Figure 6, at the end of treatment, the mock F8KO mice exhibited an increased serum RANKL (*p* < 0.01) along with a decreased serum OPG (*p* < 0.01), which caused a significant increase of the serum RANKL/OPG ratio in the F8KO mice compared to the WT mice (*p* < 0.05). With the KP treatment, the serum RANKL decreased (Figure 6A) and OPG increased (Figure 6B) in F8KO mice receiving different doses of KPs, and the combined effect led to a significant reduction in the serum RANKL/OPG ratio compared to the mock (*p* < 0.05; Figure 6C).

#### 3.5.3 Effects of KP Treatment on Serum Proinflammatory Cytokines

As shown in Figure 6D, the serum IL-6 increased significantly in the mock F8KO mice compared to the WT at the end of treatment (*p* < 0.01). Oral administration of KPs reduced the IL-6 level in the F8KO mice, especially for the group with high-dose KP treatment (*p* < 0.05 vs mock, Figure 6D). Other proinflammatory cytokines, such as IL-1α, IL-1β, and TNF-α, were also measured, but the changes were not significant (data not shown).

#### 3.5.4 Effects of KP Treatment on Osteoclastogenesis

**FIGURE 7:**
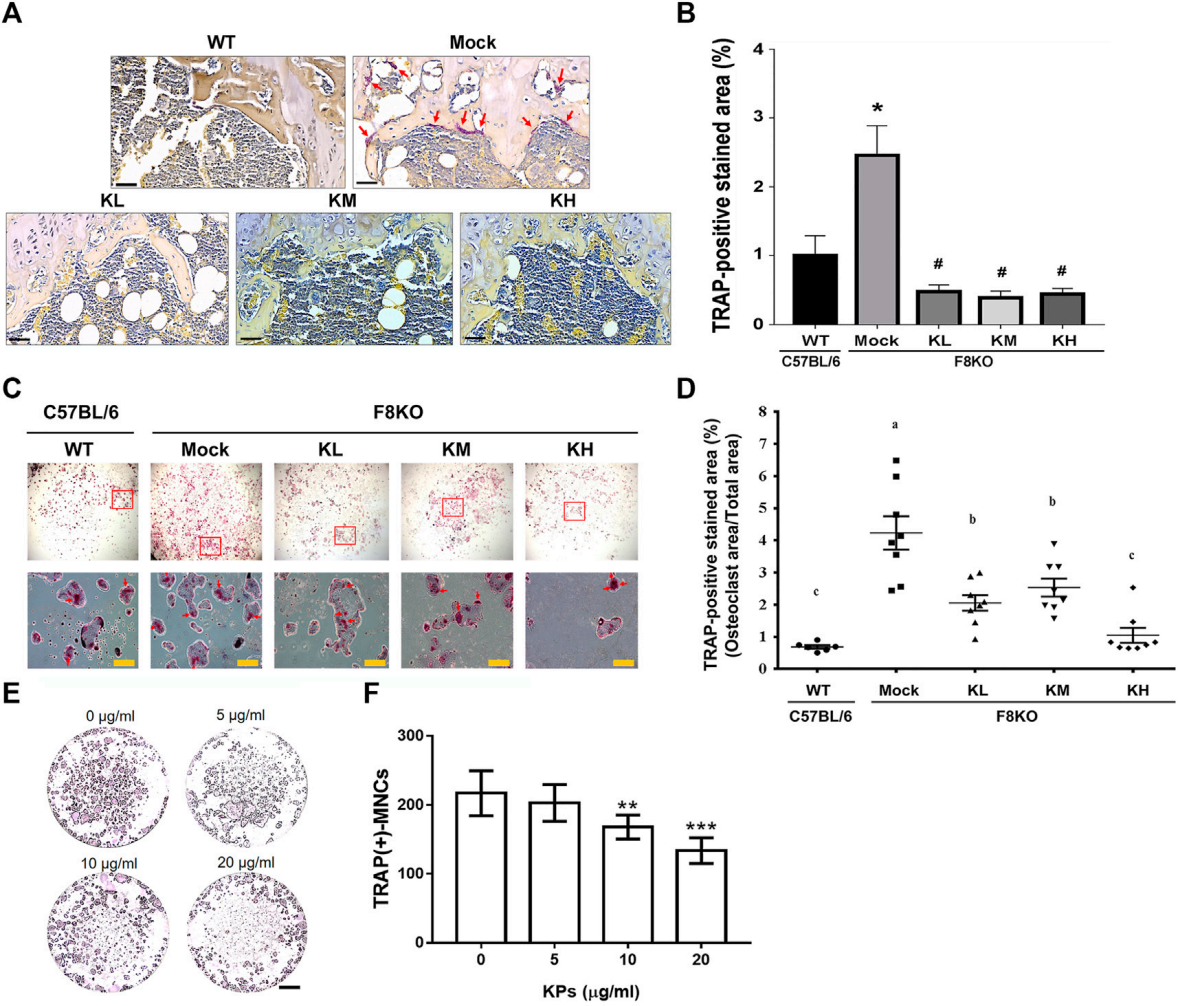
Effects of KP treatment on osteoclast differentiation. **(A)** TRAP staining of the paraffin-embedded femoral tissue sections from each group. The purple-colored TRAP-positive osteoclasts were indicated with red arrows in mock group. Scale bar = 30 µm. **(B)** TRAP-positive stained areas in each group were quantitated and compared using ImageJ software. *p < 0.05 vs WT group; ^#^ *p* < 0.05 vs mock group. **(C)** TRAP-staining of mature osteoclasts differentiated from the flushed bone marrow cells with the stimulation of M-CSF and RANKL for 14 days. The multinucleated osteoclasts in squares were magnified and indicated by red arrows. **(D)** TRAP-positive stained areas in each group were quantitated and compared using ImageJ software. *p < 0.05, **p < 0.01 vs WT group; ^#^ *p* < 0.05, ^##^ *p* < 0.01 vs. mock group. **(E)** KP treatment inhibits RANKL-induced osteoclastogenesis in the culture of bone marrow macrophages (BMMs). To stimulate osteoclast differentiation, BMMs were stimulated with M-CSF (25 ng/ml) and RANKL (50 ng/ml) in the presence of various concentrations of KPs. TRAP staining was performed at the 5^th^ day of incubation. **(F)** The TRAP-positive multinucleated cells with the number of nucleus ≥3 were considered as mature osteoclasts. **p < 0.01, ***p < 0.001 vs. 0 μg/ml of KPs; Scale bar = 1 mm.

TRAP staining was performed to examine the contents of mature osteoclasts in the paraffin-embedded femur sections from each group. As shown in Figures 7A,B, the TRAP-positive mature osteoclasts were stained in a purple-colored appearance, which were found abundant in the metaphyseal regions of the distal femur sections from the mock F8KO mice, but not apparent in the femur sections from the WT and the KP-treated groups, suggesting that KP treatment caused the inhibition of osteoclastogenesis. We performed *in vitro* osteoclast differentiation using flushed bone marrow cells from each group by stimulating with M-CSF and RANKL. As shown in Figure 7C, the TRAP-positive stained areas accounted for approximately 4.24% in the mock group, which represented an increase of 84.2% compared to the WT group (0.69% on average) (*p* < 0.01; Figure 7D). With the KP treatments, the TRAP-positive areas were significantly reduced to 2.06% in the KL group (*p* < 0.05), 2.54% in the KM group (*p* < 0.05), and 1.05% in the KH group (*p* < 0.01), which corresponded to 51.6, 40.7, and 73.1% reductions compared to the mock group (Figure 7D), respectively, suggesting that oral administration of KPs significantly inhibited osteoclastogenesis. Furthermore, we also performed a similar experiment using primary BMMs to verify the inhibitory effect of KPs on *in vitro* RANKL-induced osteoclastogenesis (Figure 7E). As shown in Figure 7F for the quantitative data, KPs dose-dependently inhibited the formation of mature osteoclasts from bone marrow cells of macrophage lineage.

## 4 Discussion

In the present study, the potential therapeutic effects of KPs on hemophilia-induced osteoporosis were investigated in an F8KO mouse model. The results revealed that KP treatment restored the femoral trabecular BMD, the trabecular architecture of the femoral bone and the 4^th^ lumbar vertebrae, and the mechanical properties of cortical bone in a dose-dependent manner. In addition, oral administration of KPs inhibited bone resorption by reducing the serum RANKL/OPG ratio and proinflammatory IL-6 levels in the F8KO mice and inhibiting osteoclastogenesis from femoral mesenchymal stem cells in an *in vitro* culture. Therefore, this study suggests that KPs can be used as a complementary or adjuvant therapy for the treatment of osteoporosis resulting from hemophilia.

F8KO mice, which contain only <1% coagulation activity, are an ideal animal model for examining the direct effect of factor VIII deficiency on bone regeneration and are also useful for the assessment of potential anti-osteoporotic therapies in PWH. Previous studies demonstrated that F8KO male mice exhibited lower femoral BMD and cortical thickness than their WT littermates at the age of 18–20 weeks, and these biological changes led to a weakened bone strength to resist fracture (Liel et al., 2012; Recht et al., 2013). Before treatment with KPs, we analyzed the femur bones of the F8KO and WT male mice, and our results confirmed that F8KO mice spontaneously developed osteoporosis at 20 weeks of age (Figure 1). Moreover, a recent study compared the bone status of total FVIII gene knockout mice (F8^TKO^), a new animal model of severe hemophilia, with their WT littermates (Weitzmann et al., 2019). This study also demonstrated that F8^TKO^ mice exhibited significant bone loss at 6 months of age and revealed a sexual dimorphism in the mechanism driving bone loss in male and female F8^TKO^ mice.

Low BMD or osteoporosis and the resultant bone fractures are prevalent age-related comorbidities in PWH, both in adults and children (Barnes et al., 2004; Wallny et al., 2007; Iorio et al., 2010). A 10-year single institutional retrospective cohort study (*n* = 382 PWH) exhibited a significantly higher relative risk (RR) of bone fracture in PWH than in the general population (*p* < 0.0001; RR: 10.7, 95% confidence interval (CI): 8.2–14.1) (Gay et al., 2015). Additionally, this retrospective study also indicated that the RR of fracture was positively correlated with the severity of hemophilia, with patients with severe hemophilia showing an increased RR of fracture compared to those with mild or moderate hemophilia (*p* < 0.05). An earlier single institutional cross-sectional study (*n* = 88) indicated that low BMD became more prevalent with the increasing severity of hemophilia in PWH <50 years, and a large proportion of PWH ≥50 years (no association with the severity of hemophilia) had osteoporosis; thus, adult PWH ≥50 years should receive routine osteoporosis detection (Kempton et al., 2014). Immune tolerance induction (ITI) is routinely used for the treatment of hemophilia with an inhibitor in PWH; however, such a therapeutic regimen has been reported to facilitate the reduction of BMD in patients with an inhibitor (Rezaieyazdi and Mansouritorghabeh, 2020). Few studies have evaluated the effects of anti-osteoporotic treatment in PWH, except ibandronate, a common bisphosphonate used for the treatment of postmenopausal osteoporosis (Anagnostis et al., 2013). This finding urges us to evaluate the effect of KPs on the treatment of hemophilia-induced osteopenia or osteoporosis in an F8KO murine model.

In our previous study, we demonstrated the potential of KPs in the prevention of postmenopausal osteoporosis in ovariectomized (OVX) rats (Chen et al., 2015). To further understand the therapeutic effect of KPs on the treatment of hemophilia-induced osteoporosis, male F8KO mice at 20 weeks of age were orally administered with low-, medium-, and high-dose KPs for 8 weeks. At the end of the experiment, the lost bone mass in the F8KO mice, as indicated by histological and 3D μ-CT images, was partially or completely restored by the KP treatment, especially in the high-dose KP-treated group (Figures 2, 3). The morphological alterations in femoral trabeculae in response to the KP treatment were consistent with the improved BMD and microarchitecture parameters, with the treatment increasing trabecular bone volume (Tb.BV/TV) and trabecular number (Tb.N), and reducing trabecular separation (Tb.Sp). We also examined the effect of KP treatments on the morphological and mechanical changes of the femoral cortical bone. By mid-shaft femur analysis (Supplementary Figure S2), we found that the thickest region of the transverse cortical bone increased significantly in all F8KO mice, but the median and the thinnest parts remained indifferent in all mice. In addition, the *x*-axis width of the transverse cortical bones (*x*-axis) decreased in all F8KO mice, but the *y*-axis width of the transverse cortical bones and the lengths of longitudinal cortical bones remained indifferent in all mice. Although KP treatment did not cause significant changes in cortical bones, it significantly improved the mechanical properties of hardness and elastic modulus in femoral cortical bones (Figure 4). Furthermore, we also identified anti-osteoporotic effect of KPs on the 4^th^ lumbar vertebra, which exhibited increased Tb.N and decreased Tb.Sp (Supplementary Figure S1). These data suggest that KP treatment can enhance bone quality and thus reduce the risk of fracture in F8KO mice or patients with hemophilia.

Biochemical biomarkers of bone turnover can be used to reflect the metabolic status of bone remodeling and provide useful information for therapy monitoring purposes during osteoporosis treatments. These biomarkers are generally divided into two categories representing bone formation and bone resorption. CTX-1 is a biomarker of bone resorption and its serum level is highly correlated with osteoclastic activity. In the present study, we found that CTX-1 elevated in untreated male F8KO mice, suggesting the lack of coagulation Factor VIII promote bone resorption, while the treatment of KPs resists the conversion of serum CTX-1. Moreover, previous study of compared the serum CTX-1 of F8^TKO^ mice with their WT littermate and indicated that CTX-1 elevated significantly only at elder female F8^TKO^ mice but remained unchanged at both young and elder male F8^TKO^ mice (Weitzmann et al., 2019). ALP and OC are two biomarkers of bone formation. Present study exhibited that the untreated F8KO mice (28 weeks old) showed a higher serum OC along with a lower serum ALP than WT mice; however, those mice treated with KPs showed a significant seroconversion of OC and ALP at the end of treatment. ALP and OC have been compared between 20-week-old F8KO and WT mice previously (Recht et al., 2013), but no significant differences have been reported. Different to the present study, all F8KO mice used in previous study were male offspring of WT males and F8KO heterozygote females (Recht et al., 2013). Another earlier study reported that 14-week-old female F8KO mice exhibited decreased OC in the collected bone marrow serum from flushed femur and tibia compared with the WT mice of the same age (Aronovich et al., 2013). These contradictory results may be caused by differences in ages, gender and genetic background of F8KO mice.

To further understand the effects of KPs on the regulation of bone metabolism in the F8KO mice, the serum levels of RANKL and OPG were measured. It has been largely reported that the RANKL/RANK/OPG signaling pathway plays a key role in the control of osteoclastogenesis (Mafi Golchin et al., 2016; Kenkre and Bassett, 2018). RANKL is the ligand of RANK on osteoclast precursors. RANKL/RANK binding induces downstream signaling pathways to regulate the expression of osteoclast genes and to drive further differentiation of osteoclast precursors into mature osteoclasts. OPG, a soluble decoy receptor of RANKL, can inhibit osteoclast differentiation by preventing the binding of RANK to its ligand RANKL. Thus, the circulating RANKL/OPG ratio reflects osteoclast activities during bone metabolism. In this study, we found that the RANKL level increased along with a decrease in OPG, which resulted in a higher RANKL/OPG ratio in the mock F8KO mice than the WT mice, suggesting that increased osteoclastogenesis was activated and that the bone remodeling balance was inclined toward bone resorption in F8KO mice. However, the KP treatment led to decreased RANKL and elevated OPG levels, and the combined effect eventually resulted in a decrease in the RANKL/OPG ratio.

Osteoclastogenesis is also regulated by circulating proinflammatory cytokines, such as TNF-α, IL-1, and IL-6. We did not identify significant alterations in TNF-α and IL-1 levels, although we found that the KP treatment tended to decrease the IL-6 levels in the F8KO mice. IL-6 is secreted by peripheral macrophages and osteoblasts, stimulates osteoclast formation and induces bone resorption (Ishimi et al., 1990). Additionally, we also found that the KP treatment resulted in decreased TRAP activity in primary osteoclast cultures, as indicated by the decreased TRAP-stained areas shown in Figure 7. These data suggested that KP treatment inhibited osteoclastogenesis in F8KO mice and thus resulted in the inhibition of bone resorption. The underlying molecular mechanisms of KPs in regulating the activities of osteoblasts and osteoclasts during bone remodeling require more detailed cellular experiments to understand the full picture.

## 5 Conclusion

In summary, we demonstrated that oral administration of KPs can elicit a therapeutic effect on osteoporosis treatment in hemophilic mice due to FVIII deficiency. The therapeutic effect of KPs is associated with their ability to inhibit osteoclastogenesis and bone resorption by decreasing the serum RANKL/OPG ratio and the secretion of proinflammatory cytokines such as IL-6. Oral administration of KPs is generally safe and more cost-effective than many anti-osteoporotic agents. Therefore, the use of KPs may have potential as a complementary or adjuvant therapy for the long-term management of bone health in patients with hemophilia.

## Data Availability

The original contributions presented in the study are included in the article/Supplementary Material, further inquiries can be directed to the corresponding author.
