# Supplementary figures and images for: Therapeutic Effects of Kefir Peptides on Hemophilia-Induced Osteoporosis in Mice With Deficient Coagulation Factor VIII
Source: Front Cell Dev Biol. 2022 Feb 18;10:794198. doi: 10.3389/fcell.2022.794198 (PMC8894723; doi:10.3389/fcell.2022.794198)

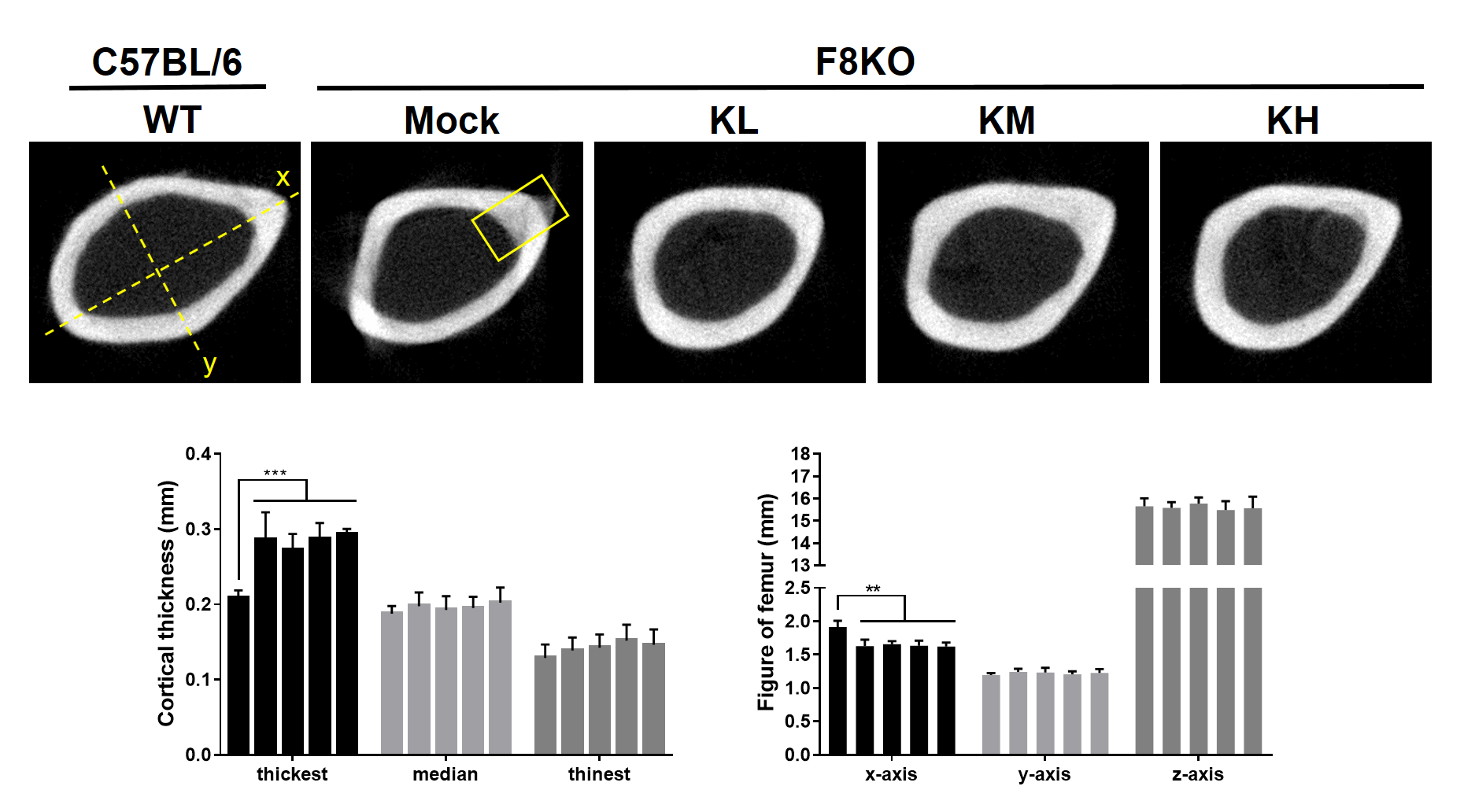

Supplement: Supplementary file 1 [file Image2.TIF]

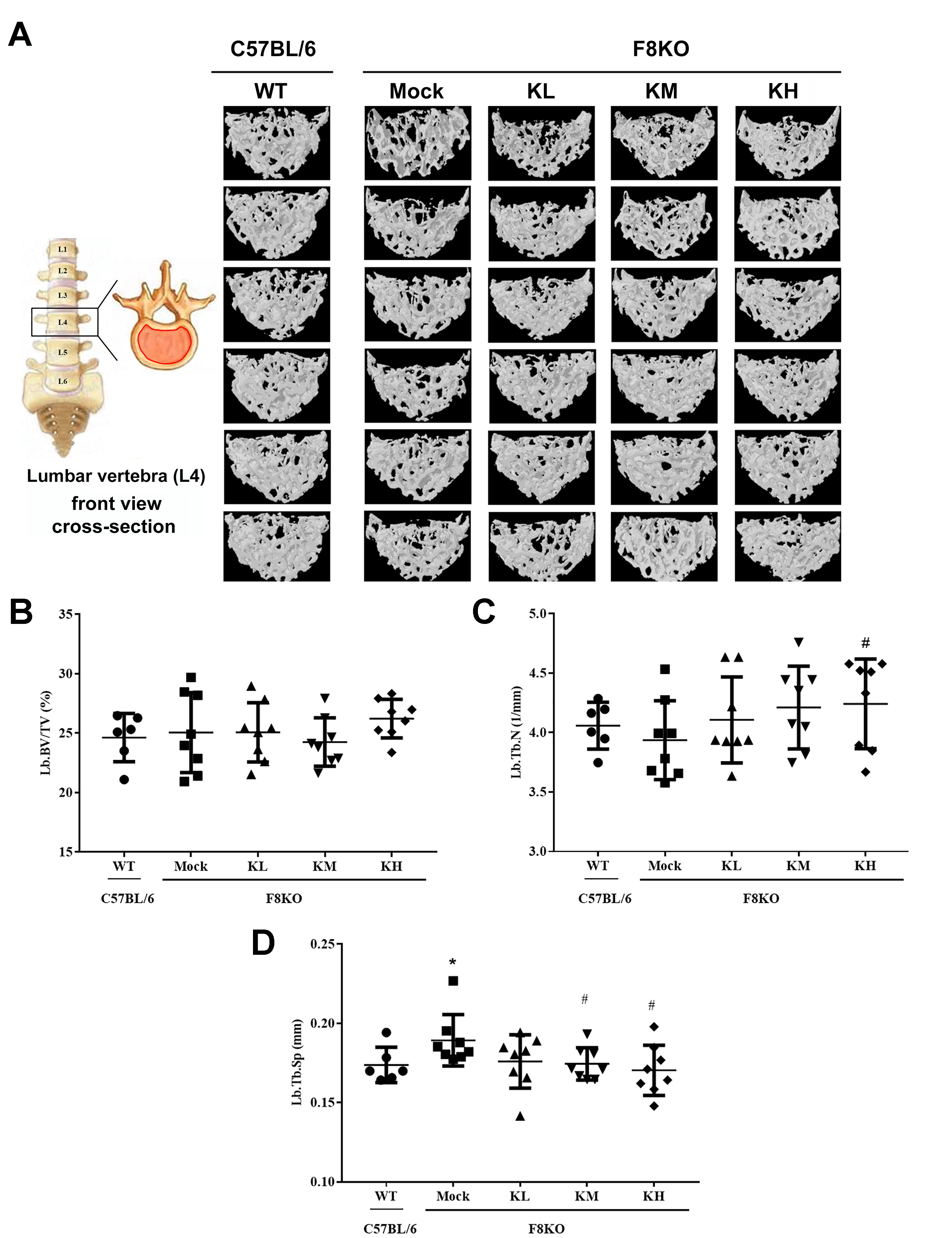

Supplement: Supplementary file 2 [file Image1.TIF]
